# Interleukin-10 Production by Th1 Cells Requires Interleukin-12-Induced STAT4 Transcription Factor and ERK MAP Kinase Activation by High Antigen Dose

**DOI:** 10.1016/j.immuni.2009.05.012

**Published:** 2009-08-21

**Authors:** Margarida Saraiva, Jillian R. Christensen, Marc Veldhoen, Theresa L. Murphy, Kenneth M. Murphy, Anne O'Garra

**Affiliations:** 1 Division of Immunoregulation, MRC National Institute for Medical Research, The Ridgeway, London NW7 1AA, UK; 2 Division of Molecular Immunology, MRC National Institute for Medical Research, The Ridgeway, London NW7 1AA, UK; 3 Department of Pathology and Centre for Immunology, Howard Hughes Medical Institute Washington University School of Medicine, St. Louis, MO 63110, USA

**Keywords:** MOLIMMUNO

## Abstract

CD4^+^ T cells producing interleukin-10 (IL-10) and interferon-γ (IFN-γ) are reported in chronic infections. However, the signals that direct the development of IL-10-producing T helper 1 (Th1) cells are undefined. We showed that development of IL-10-producing Th1 cells required high T cell receptor (TCR) ligation, sustained ERK1 and ERK2 MAP kinases phosphorylation, and IL-12-induced STAT4 transcription factor activation. Repeated TCR triggering led to enhanced IL-10 production by Th1 cells, and continued IL-12 action and high-dose TCR signaling were required for the development and maintenance of IL-10-producing Th1 cells. Although Th1, Th2, and Th17 cells require the activation of distinct STATs for their differentiation, activation of ERK1 and ERK2 was a common requirement for production of IL-10 by all Th cell subsets. IL-10 expression also correlated with *c-maf* expression. Despite having distinct functions in protection against pathogens, all Th cells share the important task of controlling overexuberant immune responses by means of IL-10 production.

## Introduction

Interleukin-10 (IL-10) is a cytokine with broad anti-inflammatory properties that inhibits macrophage and dendritic cell (DC) function (Moore et al., 2001). IL-10 limits the immune and inflammatory responses to pathogens and gut flora and prevents damage to the host (Moore et al., 2001; O'Garra and Vieira, 2004), but when dysregulated may result in chronic infection (Brooks et al., 2006; Ejrnaes et al., 2006; Moore et al., 2001). IL-10 is expressed by T helper 2 (Th2) cells, B cells, DCs, and macrophages (Moore et al., 2001), and also by Th1 cells (Anderson et al., 2007; Assenmacher et al., 1994; Del Prete et al., 1993; Gerosa et al., 1996; Jankovic et al., 2007; Pohl-Koppe et al., 1998) and (reviewed in O'Garra and Vieira, 2007; Trinchieri, 2007), certain regulatory (Treg) T cells (Moore et al., 2001; O'Garra and Vieira, 2004; Roncarolo et al., 2006), and Th17 cells (Awasthi et al., 2007; Fitzgerald et al., 2007; McGeachy et al., 2007; Stumhofer et al., 2007).

In vitro human CD4^+^ and CD8^+^ T cell clones, or mouse CD4^+^ T cells that produce both interferon-γ (IFN-γ) and IL-10, can be differentiated by T cell receptor (TCR)-stimulation in the presence of IL-12 (Chang et al., 2007; Gerosa et al., 1996; Jeannin et al., 1996; Meyaard et al., 1996; Windhagen et al., 1996). Furthermore, Th1 cell clones coproducing IFN-γ and IL-10 have been isolated from bronchoalveolar lavage (BAL) of active pulmonary tuberculosis (TB) patients (Gerosa et al., 1999). IL-10 production by Th1 cells was also reported in animals infected with *Toxoplasma gondii* (Jankovic et al., 2002; Shaw et al., 2006) or with *Leishmania major* (Anderson et al., 2007) and shown to be required for regulation of the immune response in these infections (Anderson et al., 2007; Jankovic et al., 2007). The relative amounts of IL-10 and IFN-γ produced by Th1 cells may influence the balance between clearance and persistent infection with certain pathogens (Moore et al., 2001; Trinchieri, 2007), thus determining whether chronic infection or immunopathology ensues.

Th1, Th2, and Th17 cell responses differentiate along distinct signaling pathways (Glimcher and Murphy, 2000; Ivanov et al., 2007; Stockinger and Veldhoen, 2007). Th1 cell development requires signal transducer and activator of transcription (STAT)1 activation, induced by type I IFN or IFN-γ, the transcription factor T-box 21 (T-bet), and IL-12-induced STAT4 signaling, which can couple with IL-18-induced IRAK and NF-κB transcription factors to drive the high amounts of IFN-γ required to eradicate intracellular pathogens (Glimcher and Murphy, 2000). Th2 cell development, with expression of IL-4, IL-5, and IL-13, requires IL-4, STAT6, and the transcription factor GATA binding protein (GATA)-3 (Glimcher and Murphy, 2000). The development of Th17 cells requires IL-6, TGF-β, and the STAT3-dependent expression of the transcription factor RORγt (Ivanov et al., 2007; Stockinger and Veldhoen, 2007).

Th1 and Th2 cell responses can also be induced by varying the dose of antigen presented to the naive T cell by the antigen-presenting cell (APC). Whereas high doses of antigen, with sustained TCR signaling and extracellular-signal regulated (ERK) mitogen-activated protein kinase (MAPK) phosphorylation, result in Th1 cells producing IFN-γ via an IL-12-independent mechanism, low doses of antigen, with transient ERK1 and ERK2 activation, favor Th2 responses and IL-4 secretion (Constant et al., 1995; Hosken et al., 1995; Jorritsma et al., 2003; Yamane et al., 2005).

Because Th1 and Th2 cells cross regulate each other's development and function and can suppress Th17 cell responses, and all differentiate along distinct signaling pathways (Glimcher and Murphy, 2000; Stockinger and Veldhoen, 2007), IL-10 produced by all these Th cells may thus act as a feedback regulator to control the pathology associated with an overexuberant, albeit efficacious, inflammatory response. Whether IL-10 production by these different Th cell subsets is induced by independent and/or common mechanisms is unknown.

Here, we showed that in vitro differentiation of IL-10-producing Th1 cells from naive CD4^+^ T cells required IL-12-induced STAT4 signaling, strong TCR activation (high antigen dose), and sustained ERK1 and ERK2 phosphorylation. Furthermore, we showed that activation of ERK1 and ERK2 is a requirement for production of IL-10 by Th1, Th2, and Th17 cell subsets. This common but highly regulated pathway for IL-10 induction and maintenance ensures its function as a feedback loop to control damage to the host and also allows a protective response to ensue as opposed to chronic infection.

## Results

### IL-12 and High Doses of Antigen Induce the Development of Th1 Cells Producing IL-10

To study the differentiation of Th1 cells coproducing IFN-γ and IL-10, we cultured purified TCR-transgenic DO11.10 naive CD4^+^ T cells with purified DCs as APCs and increasing doses of ovalbumin peptide 323-339 (OVA). Culture with high doses of antigen for 7 days gave rise to Th1 cells expressing IFN-γ upon restimulation (Constant et al., 1995; Hosken et al., 1995), but not IL-10 (Figure S1A available online). Culture with low antigen doses under the same conditions led to the differentiation of Th2 cells, which expressed both IL-4 and IL-10 upon restimulation (Figure S1A). Culture of naive CD4^+^ T cells in an APC-free system by stimulation with anti-CD3 and anti-CD28 antibodies in the presence of IL-12 resulted in IFN-γ-producing Th1 cells, a proportion of which coproduced IL-10, as did Th2 cells resulting from culture in IL-4 (Figure S1B).

**Figure 1:**
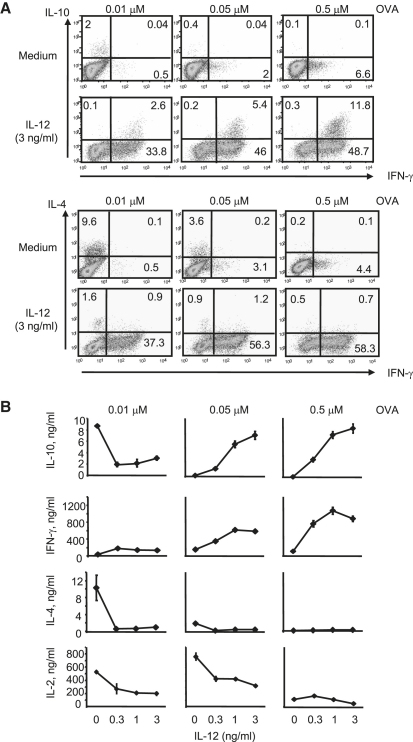
IL-12 Enhances IL-10 Production at High Antigen Dose Only Naive CD4^+^ T cells from DO11.10 TCR transgenic animals were isolated and differentiated for 1 week with splenic DCs in medium containing increasing doses of OVA in the absence (medium) or presence of IL-12, as indicated. On day 7, cells were restimulated for 4 hr with plate-bound anti-CD3 and soluble anti-CD28 and BrefeldinA and (A) stained at the single-cell level for IL-10, IFN-γ, and IL-4, or (B) stimulated similarly for 48 hr in the absence of BrefeldinA, and the culture supernatants analyzed by ELISA. Data are representative of six experiments performed; error bars in (B) refer to the standard deviation of three independent cultures.

To investigate whether the lack of IL-10 produced by Th1 cells resulted from inhibition of IL-10 production by DCs and high antigen dose or alternatively required IL-12, we cultured naive CD4^+^ T cells with increasing doses of antigen presented by DC in the presence of IL-12. At low doses of antigen, IL-12 abrogated the development of Th2 cells and induced IFN-γ expression but only low levels of IL-10 expression, suggesting that IL-12 per se was not sufficient to induce significant IL-10 production in Th1 cells (Figure 1A). Strikingly, as the antigen dose was increased, Th1 populations driven with IL-12 now contained higher numbers of IL-10-producing cells (Figure 1A) and produced more IL-10 protein upon restimulation (Figures 1A and 1B). Thus, the development of Th1 cells producing IL-10 required both IL-12 and high doses of antigen. Th1 cells differentiated to produce large amounts of IL-10 and IFN-γ and lost their capacity to produce IL-2 (Figure 1B). Because the presence of IL-12 reduced the proliferation of CD4^+^ T cells at both high and low antigen doses (Table S1), and only the former showed IL-10 production, the development of high IL-10-producing cells is most likely not related to limited IL-2. Naive CD4^+^ T cells from DO11.10/recombination-activating gene 1 (Rag1)-deficient animals, cultured with high doses of antigen in the presence of IL-12, also resulted in IL-10 expression by Th1 cells, showing that this expression was not dependent on the presence of effector or memory T cells or Treg cells (Figure S2). Although it has been suggested that TGF-β can induce IL-10 in CD4^+^ T cells (Kitani et al., 2003; Schiott et al., 2000), we found that in developing Th1 and Th2 cells this was not the case (data not shown). In fact, neutralization of TGF-β led to increased IL-10 production by both T cell subsets (Figure S3). Thus, the development of IL-10-producing Th1 cells only depended on the presence of IL-12 together with high antigen dose and not on other soluble factors such as IL-2 or TGF-β or on the presence of other T cell types.

### IL-10 Production by Th1 Cells Is Dependent on STAT4 but Not on STAT6, IFN-γ, or IL-4 Signaling

**Figure 2:**
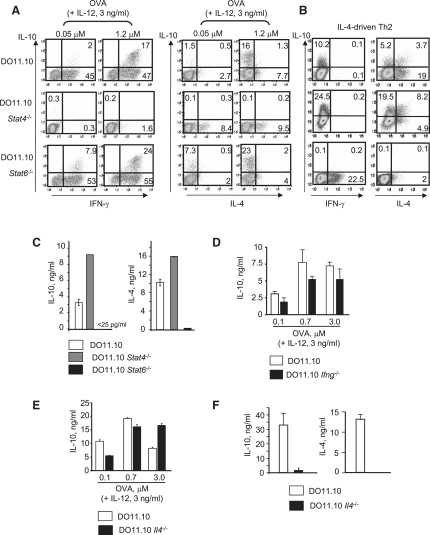
IL-10 Production by Th1 Cells Requires IL-12-Induced STAT4 Activation CD4^+^ T cells from DO11.10 animals on a (A, B, and C) WT, *Stat4*^−/−^, or *Stat6*^−/−^ background, (D) *Ifnγ*^−/−^ background, or (E and F) *Il4*^−/−^ background were isolated and differentiated as in Figure 1. As shown in (B), Th2-driven cells were differentiated for 1 week in medium containing IL-4 (10 ng/ml) and anti-IL-12 (10 μg/ml) plus 1 μM OVA; or (C and F) 0.05 μM OVA alone (low-antigen-dose-driven Th2 cells). On day 7, cells were restimulated and IL-10, IFN-γ, and IL-4 production were detected by ICS or by immunoassay as in Figure 1. Represented is one of three experiments performed. Detection of cytokines by ELISA in the supernatants of cultures restimulated for 48 hr (data not shown) confirmed the results observed for ICS in (A) and (B). Error bars refer to the standard deviation of replicate values of three independent cultures.

To further elucidate the mechanisms required for the development of Th1 cells producing IL-10, we investigated the role of STAT4, one of the signaling pathways activated by IL-12 (Murphy et al., 2000). Naive CD4^+^ D011.10 T cells deficient in STAT4 (Ouyang et al., 1998) were cultured in the presence of IL-12 and OVA. Again, IL-10-producing Th1 cells were differentiated at the high antigen dose in the presence of IL-12 in DO11.10 T cells (Figure 2A). In contrast, in the absence of STAT4, the percentage of cells expressing IFN-γ was dramatically diminished as expected and resulted in an increase in the percentage of cells expressing IL-4, but not IL-10 (Figure 2A), suggesting that STAT4 contributes to IL-10 expression by Th1 cells.

Because IL-10 expression is associated with an IL-4-induced Th2 cell phenotype, we investigated whether the differentiation of the IL-10-producing Th1 cells depended on signaling through the IL-4 receptor via STAT6 activation (Glimcher and Murphy, 2000; Murphy et al., 2000). The absence of STAT6 did not impair the differentiation of IL-10-producing Th1 cells in the presence of IL-12 and OVA (Figure 2A). In fact, a higher percentage of STAT6-deficient cells compared with WT cells produced both IL-10 and IFN-γ (Figure 2A), which may be the result of the loss of Th2 cell control over a Th1 cell response. As expected, lack of STAT6 abrogated both IL-4 and IL-10 production by T cells developed with IL-4 or with low antigen dose (Figures 2B and 2C). However, in the absence of STAT4 signaling, IL-10 and IL-4 production by Th2 cells was if anything increased (Figures 2B and 2C). Thus, in contrast to what was observed under Th1 conditions, IL-10 expression by Th2 cells depended on STAT6, but not on STAT4, signaling (Figures 2B and 2C).

To investigate whether the inability of STAT4-deficient T cells to produce IL-10 might be due to the absence of IFN-γ, as suggested before (Shaw et al., 2006), we differentiated DO11.10 or DO11.10 IFN-γ-deficient naive CD4^+^ T cells in the presence of IL-12 and increasing doses of OVA. The secretion of IL-10 as induced by high antigen dose, and IL-12 was not affected by an absence of IFN-γ (Figure 2D), showing that the expression of IL-10 by Th1 cells is independent of IFN-γ. In the absence of IFN-γ, we observed an increase in the secreted IL-4 as expected (data not shown).

We also tested for any potential role of IL-4 in the development of Th1 cells producing IL-10 by culturing DO11.10 or DO11.10 IL-4-deficient naive CD4^+^ T cells with IL-12 and increasing doses of antigen. As observed in the absence of STAT6 (Figure 2A), IL-4 deficiency had no effect on the development of Th1 cells producing IL-10 (Figure 2E), but compromised the development of Th2 cells producing IL-10 (Figure 2F). Thus, our data suggested that IL-10 production by Th1 or Th2 cells was dependent on the specific signaling pathways required for their differentiation, given that STAT4 is required for the induction of IL-10 production by Th1 cells and STAT6 for Th2 cells.

### High Antigen Doses and STAT4 Are Required for the In Vivo Generation of IL-10-Producing Th1 Cells

**Figure 3:**
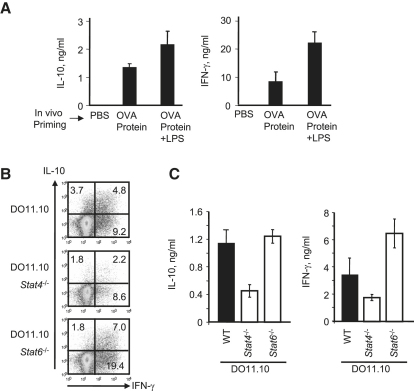
In Vivo Generation of IL-10-Producing Th1 Cells Requires High Antigen Dose, Innate Immune Signals and STAT4 (A) Spleen cell suspensions from DO11.10 animals were injected into BALB/c recipients. Cells were isolated from the inguinal lymph nodes of mice injected with PBS, OVA protein (5 mg) plus or minus LPS (5 μg) and restimulated in vitro with OVA for 48 hr. IL-10 and IFN-γ production was analyzed by ELISA. Represented is the mean ± SD of groups of three animals each. Spleen cell suspensions from DO11.10 WT (ten animals), *Stat4*^−/−^ (nine animals), or *Stat6*^−/−^ (ten animals) were transferred into BALB/c recipients, which were then injected with OVA protein and LPS (5 mg and 5 μg, respectively). Cells recovered as above were restimulated in vitro, for 24–48 hr with the last 6 hr in the presence (for ICS, B) or absence (for ELISA, C) of BrefeldinA. IL-10 and IFN-γ expression was detected by ICS and represented within the CD4^+^ KJ1-26^+^ T cells population. (B) We obtained the plots represented by merging the plots from each mouse within one group. Two independent experiments confirmed the result showed and the mean percentage of IL-10/IFN-γ producers ± SD was 3.95 ± 1.15 and 2.25 ± 0.68; 4.29 ± 1.36 and 7.11 ± 1.86; and 3.82 ± 0.96 and 10.19 ± 2.25 for DO11.10 WT, *Stat4*^−/−^, and *Stat6*^−/−^, respectively. (C) Error bars refer to the standard deviation of replicate values on two independent experiments.

To address the mechanisms regulating IL-10 production by Th1 cells in vivo, we transferred DO11.10 cells into BALB/c recipient mice and immunized the recipients with very high doses of OVA-protein with or without added lipopolysacharide (LPS). T cells were recovered from the inguinal lymph nodes 3 days after priming and restimulated in vitro with OVA peptide for 48 hr. This in vivo immunization induced IL-10 and IFN-γ production, and the amount of IL-10 production was enhanced by addition of LPS in the immunization (Figure 3A) and with higher doses of OVA (3 μM versus 1 μM, data not shown). To test the role of STAT4 and STAT6 signaling in the in vivo development of IL-10-producing Th1 cells, we transferred STAT4- or STAT6-deficient or WT DO11.10 cells into recipient BALB/c mice and immunized with OVA-protein plus LPS as before. In vivo expression of both IL-10 and IFN-γ was markedly reduced but not completely abrogated in the absence of STAT4 signaling (Figures 3B and 3C), suggesting the existence of compensatory mechanisms that were absent in the in vitro system. Signaling through STAT6 had no effect on IL-10 production by Th1 cells as shown by intracellular cytokine staining (ICS) and by immunoassay in STAT6-deficient T cells (Figures 3B and 3C).

### IL-10 Production Is Maintained by High TCR Signal Strength and IL-12

**Figure 4:**
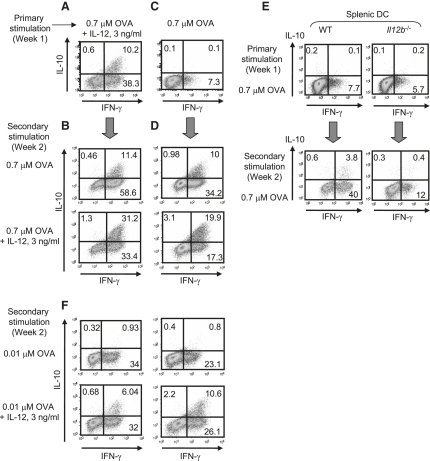
High Antigen Dose and IL-12 Are Required for Maximal Production of IL-10 by Th1 Cells CD4^+^ T cells from DO11.10 TCR transgenic animals were cultured for 1 week with a high (0.7 μM) dose of OVA and DC in the (A and B) presence or (C) absence of IL-12 and subsequently washed, counted, and recultured with a high (0.7 μM; B, D, and E) or low (0.01 μM; F) dose of OVA and DC in the absence or presence of IL-12 as indicated. CD4^+^ T cells from DO11.10 TCR transgenic animals were cultured with a high (0.7 μM) dose of OVA and WT or *Il12b*^−/−^ DCs in the absence of added IL-12 for 2 weeks (E). At the end of the first (A, C, and E) and the second week (B, D, E, and F), cells were restimulated with anti-CD3 and anti-CD28, and IFN-γ and IL-10 cytokine expression were detected by ICS. Represented is one of three experiments performed.

We next investigated whether repeated strong TCR activation is a compensatory signal for IL-12-induced STAT4 signaling in the induction of IL-10 in Th1 cells. For this, CD4^+^ T cells were differentiated for 2 consecutive weeks with high antigen doses in the presence or absence of IL-12 throughout (Figures 4A–4D). High antigen dose and IL-12 cooperated to induce maximal IL-10 production (Figures 4A and 4B), given that this combination resulted in the highest numbers of IL-10-producing Th1 cells. Repeated high antigen dose stimulation in the absence of exogenously added IL-12 resulted in the production of IL-10 by Th1 cells, suggesting that repeated strong TCR triggering may overcome the need for IL-12 for IL-10 induction (Figures 4C and 4D). However, IL-10 induction under these conditions was abrogated when IL-12p40-deficient DCs were used as APCs (Figure 4E). Thus, IL-12 is essential during both primary and secondary antigenic stimulation for production of IL-10 by Th1 cells.

To determine the requirements for stability of the IL-10-producing Th1 cells, we differentiated CD4^+^ T cells for 1 week with high antigen doses with or without IL-12 (Figures 4A and 4C), washed them, and then restimulated them for an additional week with a low antigen dose, in the absence or presence of IL-12 (Figure 4F). Th1 cells induced in the first week to produce IL-10 by culture with high antigen doses and IL-12 lost their ability to express IL-10 when recultured with low doses of OVA, which could be compensated for, to some extent, by addition of IL-12 to the secondary cultures (Figure 4F), again suggesting that antigen dose and IL-12 signals cooperate for the induction of IL-10. Finally, DO11.10 CD4^+^ cells that were exposed to low doses of antigen and IL-12 during the primary differentiation phase produced high amounts of IFN-γ but little IL-10, but they could be induced to produce IL-10 when both high antigen dose and IL-12 were present during the recall phase (Figure S4). Thus, high antigen dose and IL-12 are required for sustaining the induction of IL-10 production by Th1 cells.

### IL-10 Production by Th1 Cells Requires ERK1 and ERK2 Activation

**Figure 5:**
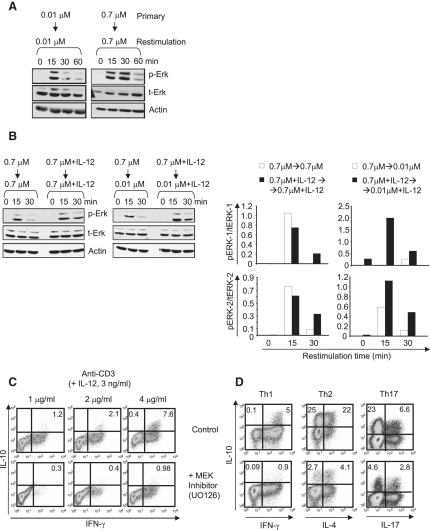
IL-10 Production by Th1, Th2, and Th17 Cells Requires ERK1 and ERK2 Activation CD4^+^ T cells from DO11.10 TCR transgenic animals were cultured with a high (0.7 μM) or low (0.01 μM) dose of OVA and DC in the absence or presence of IL-12. On day 3, cells were harvested, rested for 5 hr in low (1%) serum medium, and restimulated with high (0.7 μM) or low (0.01 μM) antigen dose, in the (A and B) absence or in the (B) presence of IL-12, for the indicated time points. Cellular lysates were prepared, proteins were separated by SDS-PAGE, and phospho(p)- or total(t)-ERK1 and ERK2 were detected by immunoblotting. Actin was included as a loading control (A and B). CD4^+^ T cells from BALB/c animals were isolated and cultured for 7 days in the presence of increasing doses of plate-bound anti-CD3, soluble anti-CD28, and IL-12 (3 ng/ml), in the absence (control, with DMSO) or presence of U0126 (2.5 μM) (MEK Inhibitor). As shown in (C), on day 7, cells were restimulated as above and the expression of IL-10 and IFN-γ was detected by ICS. Cytokine production in the supernatants of 48 hr restimulated cultures was analyzed by ELISA and confirmed the ICS results (data not shown). As shown in (D), CD4^+^ T cells from BALB/c animals were isolated and cultured for 5 days in the presence of plate-bound anti-CD3, soluble anti-CD28 and one of the following combinations: IL-12 (3 ng/ml) plus anti-IL-4 (20 μg/ml), for Th1; IL-4 (10 ng/ml) plus anti-IL-12 (10 μg/ml), for Th2; or IL-6 (50 ng/ml), TGF-β (1 ng/ml) and IL-1 (10 ng/ml), for Th17, all in the absence (control, with DMSO) or presence of PD184352 MEK Inhibitor, as in (C). On day 5, cells were restimulated with PdBU and ionomycin, and the expression of IL-10, IFN-γ, IL-4, and IL-17 was detected by ICS. Represented is one of three (A, B, C, and F) or one of four (D and E) experiments performed.

Our data showed that the maintenance of IL-10 induction in Th1 cells required stimulation with high antigen dose, which to some extent could be compensated for by the addition of IL-12. Signaling through the TCR with high doses of antigen induced stronger ERK1 and ERK2 activation than that induced by low antigen dose, not only in naive CD4^+^ T cells (data not shown) as previously demonstrated (Jorritsma et al., 2003) but also in CD4^+^ T cells restimulated with the same high and low antigen doses (Figure 5A). Although the apparent peak and amount of ERK1 and ERK2 activation varied slightly between experiments, a consistent finding was that high antigen dose differentiated Th1 cells always showed enhanced and prolonged ERK1 and ERK2 activation in the presence of IL-12, regardless of whether they were restimulated with high or low antigen dose (Figure 5B).

We then investigated whether ERK1 and ERK2 activation was required for the induction of IL-10 in Th1 cells by using U0126 (Figure 5C), a compound that blocks downstream ERK activation. To ensure that only T cell signaling was being affected by U0126, we used an APC-free system in which the T cells were differentiated in the presence of increasing doses of anti-CD3 and a constant amount of IL-12. As in the APC-driven cultures, stronger TCR stimulation together with IL-12 led to higher percentages of cells producing both IL-10 and IFN-γ after 1 week of culture (Figure 5C). Addition of U0126 to the cultures abrogated the production of IL-10 at all doses of anti-CD3 (Figure 5C). Because U0126 inhibits the MEK5-catalyzed activation of ERK5, as well as the MEK1- and MEK2-catalyzed activation of ERK1 and ERK2 (Bain et al., 2007; Mody et al., 2001), we also used the more specific, structurally unrelated MEK1 and MEK2 inhibitor PD184352 at concentrations in which it inhibits MEK1 and MEK2 but not MEK5 (Bain et al., 2007; Mody et al., 2001). PD184352 caused a similar inhibition of IL-10 production by Th1 cells in a dose-dependent fashion (Figure 5D and Figure S5A). Upon addition of inhibitors to other signaling pathways, including a p38 MAPK inhibitor, SB203580, or the GSK3β inhibitor, CT99021 (Bain et al., 2007), no effect on IL-10 production was observed (Figure S5B). Our data thus suggested that IL-10 production by Th1 cells in response to high antigen dose and IL-12 requires ERK1 and ERK2 signaling, but not the activation of the p38 or the GSK3β pathways.

### IL-10 Production by Th2 and Th17 Cells Also Requires ERK1 and ERK2 Activation

To address whether IL-10 production by Th2 and Th17 cells was also dependent on ERK1 and ERK2 activation, we differentiated these cells with anti-CD3 and anti-CD28 in the absence of APCs (Shoemaker et al., 2006; Veldhoen et al., 2009; Veldhoen et al., 2006), in the presence or absence of the MEK inhibitor (PD184352). We showed that ERK1 and ERK2 activation is a common pathway required for induction of IL-10 in different Th cell subsets because IL-10 production by both Th2 and Th17 cells was markedly inhibited in the presence of the MEK inhibitor (PD184352) (Figure 5D and Figure S5B). In contrast, inhibitors of p38 MAPK or of GSK-3β activation did not affect the expression of IL-10 by these subsets (Figure S5B). Activation of the ERK1 and ERK2 signaling pathway is therefore a common requirement for the induction of IL-10 production by Th1, Th2, and Th17 cells.

### *c-maf* Expression Correlates with IL-10 Production in Th1, Th2, and Th17 Cells

**Figure 6:**
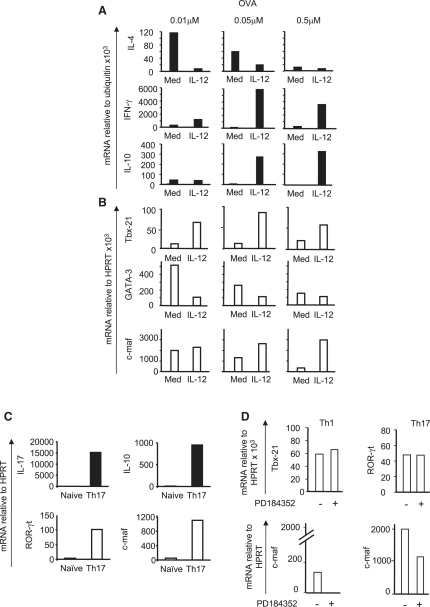
*c-maf* Is Expressed in IL-10-Producing Th1, Th2, and Th17 Cells CD4^+^ T cells from DO11.10 TCR transgenic animals were differentiated for 1 week in medium containing increasing doses of OVA plus DC in the absence (Med) or presence of IL-12, as indicated. On day 7, cells were (A) restimulated for cytokine expression or (B) not restimulated for transcription factor expression as in Figure 1. CD4^+^ T cells from C57Bl/6 animals were isolated and cultured for 5 days in the presence of plate-bound anti-CD3 (50 ng/ml), soluble anti-CD28 (50 ng/ml), IL-6 (50 ng/ml), TGF-β (1 ng/ml), and IL-1 (10 ng/ml); Th17 cells were restimulated on day 5 (for cytokine expression) or not (for transcription factor expression) as in Figure 5C. Th1 and Th17 cells were cultured as in Figure, all in the absence (−; Control), with DMSO, or presence (+) of the PD184352 MEK inhibitor. Transcription factor expression is shown in (D). Similar results were obtained from three (A, B, and C) or two experiments (D, with two doses of inhibitor or control). Cytokine and transcription factor expression were analyzed from RNA isolated from all populations by RT real-time PCR.

To investigate further the downstream factors involved in regulating IL-10 production, we differentiated DO11.10 CD4^+^ T cells with increasing doses of OVA, in the presence or absence of IL-12, and quantified the expression of cytokines and transcription factors by real-time RT-PCR. Low-dose antigen resulted in transcription of *Il4* and this was abrogated by both high antigen doses and IL-12 (Figure 6A). A low amount of transcription of *Ifn*γ was induced by IL-12 when cells were differentiated with low antigen dose, but this effect of IL-12 was markedly upregulated with increasing doses of antigen (Figure 6A). A low amount of *Il10* transcription was observed at low doses of antigen accompanying *Il4* expression (Th2 cell response), and this was abrogated by increased doses of antigen as was *Il4* expression (Figure 6A). At low doses of antigen, IL-12 had little effect to increase IL-10 mRNA expression (Figure 6A) in keeping with the protein data (Figure 1A). However, IL-12 induced a high amount of *Il10* transcription as well as *Ifnγ* expression with increased antigen doses (Figure 6A), again in keeping with the protein data (Figure 1A).

CD4^+^ T cells differentiated with increasing doses of antigen did not express high amounts of Tbx-21 (T-bet) mRNA, unless they were cocultured with IL-12 (Figure 6B). In contrast, high amounts of GATA-3 mRNA expression were only observed under Th2 cell differentiation conditions (low-dose antigen) (Figure 6B), and this expression was markedly downregulated by both increasing antigen dose and coculture in IL-12 (Figure 6B). Differentiation of T cells under low antigen dose led to expression of *c-maf*, in keeping with the Th2 cell profile (Ho et al., 1996), which was almost completely abrogated by increasing doses of antigen (Figure 6B). Interestingly, IL-12 sustained the high expression of c-Maf mRNA even at the highest antigen dose (Figure 6B). Moreover, IL-12 maintenance of *c-maf* expression required STAT4 activation (data not shown).

In Th17 cells that expressed IL-17a as well as IL-10 mRNA (Figure 6C), T-bet and GATA-3 mRNA were undetectable (data not shown), whereas that of ROR-γt was high (Figure 6C) (Ivanov et al., 2007). Th17 cells also expressed high amounts of *c-maf* (Figure 6C), confirming a recent report (Bauquet et al., 2009). c-Maf is therefore expressed in all IL-10-expressing T cell populations tested (Figures 6B and 6C) and may not be just a Th2 cell-specific transcription factor as originally thought (Ho et al., 1996). We showed also that like *Il10* expression, *c-maf* expression was inhibited in Th1 and Th17 cells in the presence of the MEK1 and MEK2 inhibitor (PD184352), whereas *T-bet* and *RORγt* expression was hardly affected (Figure 6D).

## Discussion

IL-10 expression by cells of the innate and adaptive immune systems reflects the importance of this cytokine in the tight regulation of the immune response, to minimize pathology during infection. IL-10 expression by Th1 cells has been reported to regulate the immune response in leishmaniasis and toxoplasmosis. However, in many situations, IL-10 is not produced by Th1 cells in response to antigenic stimulation. Our goal was to address the molecular signals that determine whether Th1 cells develop to produce IL-10 (Trinchieri, 2007). Here, we showed that Th1 cells required high-antigen-dose-induced ERK1 and ERK2 phosphorylation and IL-12-induced STAT-4 activation to produce IL-10. Our findings that ERK1 and ERK2 activation was a common pathway required for the production of IL-10 by Th1, Th2, and Th17 cell subsets, which differentiate along distinct pathways, such that IL-10 provides a highly regulated feedback loop to avoid the extremes of excessive inflammation or chronic infections and also allow a protective response to diverse pathogens.

In certain viral or parasitic infections (Anderson et al., 2007; Brooks et al., 2006; Ejrnaes et al., 2006), high amounts of stimulation may lead to the chronic nonhealing infection shown to be regulated by IL-10. During the course of infection, after initial triggering with antigen, T cells migrate to the tissue encountering high doses of antigen and factors produced by the innate immune response. Under these conditions, we speculate that Th1 cells will be induced to express high amounts of IL-10, in keeping with reports that IL-10-producing Th1 cells were found in CD4^+^ clones isolated from BAL but not blood of TB patients (Gerosa et al., 1999). Similarly, the immune response to a clinical isolate of *L. major*, which produces heavily infected nonhealing lesions, was found to be regulated by IL-10 derived from Foxp3^−^ Th1 cells that coproduce IL-10 and IFN-γ (Anderson et al., 2007), and the immune response during *T. gondii* infection was found also to be regulated by Foxp3^−^ Th1 cells (Jankovic et al., 2007). It is likely that IL-10 production by Th1 cells is evoked under conditions of high inflammation and antigenic stimulation, whereas regulatory CD4^+^ T cells producing IL-10 may operate to regulate the immune response under conditions in which the pathogen is clinically controlled, such as in infection with *L. major* (Friedlin strain) (Belkaid et al., 2002; Suffia et al., 2006). We now also reported that CD4^+^ T cells cultured with high antigen dose and IL-12 differentiate into canonical Th1 effector cells, which, in addition to expressing large amounts of IFN-γ and IL-10, lose their IL-2 expression as described before in certain chronic infection models (Sallusto et al., 2004). Our demonstration that loss of IL-2 is accompanied by production of IL-10 offers potential additional mechanisms whereby effector T cell responses may be dampened during chronic disease.

Using an in vivo transfer model of DO11.10 TCR transgenic cells (Castro et al., 2000), we showed that IL-10-producing Th1 cells were differentiated in the presence of high doses of OVA protein and LPS. We showed here that this induction of IL-10 in Th1 cells in vivo was markedly, but not totally, reduced in STAT4-deficient T cells as observed during *T. gondii* infection (Jankovic et al., 2002). A high antigenic activation during *T. gondii* infection or high antigen doses delivered in the presence of LPS, as seen in our system, may compensate for an absolute requirement for IL-12 in the induction of IL-10 by Th1 cells.

In our in vitro system, repeated stimulation of Th1 cells with high antigen doses allowed the development of Th1 cells producing IL-10 in an IL-12-dependent manner. IL-10 production by Th1 cells induced by high antigen dose and IL-12 was independent of IFN-γ, in keeping with previous findings (Jankovic et al., 2002). However, a role for IFN-γ in mediating IL-10 reactivation by Th1 cells during secondary infection with *T. gondii* has been suggested (Shaw et al., 2006). We have found that CD4^+^ T cells exposed to a high dose of antigen do not express IL-10 upon restimulation, but can be induced to produce IL-10 upon re-exposure to a high dose of antigen in the recall phase in the absence of added IL-12. However, this is dependent on the induction of IL-12 by antigen-presenting DCs. The combination of both high antigen dose and IL-12 resulted in the highest levels of IL-10 production and correlated with the high levels of ERK1 and ERK2 activation. The increased expression of IFN-γ observed during the secondary phase will induce increased IL-12 production by DCs and suggests that repeated high-level TCR activation feeds back to upregulate IL-12 production by DC. It is thus likely that in *T. gondii* infection in vivo (Shaw et al., 2006), the requirement for IFN-γ to induce IL-10, was for feedback upregulation of IL-12 by DCs, which in turn induced IL-10 in the Th1 cells.

Although IL-10 may be differentially regulated in Th1 and Th2 cells as has been reported (Chang et al., 2007; Wang et al., 2005), some studies suggest the existence of common pathways, but the molecular basis for these is as yet unclear. Costimulatory OX-40 signals have been shown to negatively regulate IL-10 production (Ito et al., 2005) both in Th1 and Th2 cells, whereas ICOS signaling has been suggested to induce IL-10 (Ito et al., 2007; Witsch et al., 2002) in both Th1 and Th2 cells. However, in some cases, ICOS signaling also regulates IL-4 production and Th2 responses (Greenwald et al., 2005). We now provide a common mechanism of ERK1 and ERK2 activation for the regulation of IL-10 production in Th1, Th2, and Th17 cells, although each subset differentiates along a distinct and subset-specific transcriptional pathway. This reinforces the fact that IL-10 is not a Th cell-subset-specific cytokine, but instead is produced in a tightly regulated fashion during each differentiation pathway. Of note, a role for ERK1 and ERK2 activation in the induction of IL-10 production has already been described for macrophages and DC (Agrawal et al., 2006; Hacker et al., 1999).

Differential transcriptional regulation of IL-10 in Th1 and Th2 cells has been suggested (Chang et al., 2007; Wang et al., 2005), and extensive histone acetylation of the IL-10 gene is detectable in fully polarized Th2 cells, but not Th1 cells (Chang et al., 2007). We provide evidence that IL-10 is produced in canonical Th1 cells and that its expression correlates with the expression of *T-bet* and the highest IFN-γ production, in keeping with our observations that high-dose antigen stimulation and IL-12 signaling are required for IL-10 and IFN-γ expression. It has also been shown that maintenance of IL-10 expression is conditional on IL-12 or IL-4 unless the IL-10 gene is imprinted by GATA-3 (Chang et al., 2007), which can remodel the IL-10 locus, thus explaining the highest amounts of IL-10 produced by Th2 cells (Chang et al., 2007; Shoemaker et al., 2006). We show here that high antigen dose and IL-12 drastically downregulate *Gata-3* expression, suggesting that additional factors are in place to induce IL-10 expression in Th1 cells, albeit transiently. Expression of *c-maf* was greatly diminished by high antigen doses in T cells and yet was unexpectedly maintained by IL-12 and present in Th17 cells. That *c-maf* expression is common to IL-10-producing Th1, Th2, and Th17 cells and, like IL-10, is dependent on ERK activation in Th1 and Th17 cells for its expression is of interest because c-Maf has been shown to be an essential transcription factor for IL-10 expression in macrophages (Cao et al., 2005).

In summary, we show that although Th1, Th2, and Th17 CD4^+^ T cell subsets differentiate along distinct signaling and transcriptional pathways, they can all be induced to make IL-10. ERK1 and ERK2 activation is required for IL-10 production by all these Th cell subsets. With regard to the expression of IL-10 by Th1 cells, our data provide a mechanism for how IL-10 expression is induced and then amplified and regulated by the levels of antigen and IL-12 encountered in the environment. This provides a mechanism whereby a Th1 cell responds to extrinsic signals, reflecting increased inflammation in the tissue, to tightly regulate the production of IL-10 so as to allow a protective response to eradicate a pathogen with minimal damage to the host and also prevent chronic infection. Moreover, our findings have important implications for the regulation of IL-10 production during an inflammatory Th1 response in infection and may be of relevance for the design of vaccines and for strategies in immunotherapy in infectious diseases.

## Experimental Procedures

### Mice, Cytokines, Antibodies, and Other Reagents

BALB/c DO11.10 mice transgenic for OVA-specific TCR WT or crossed back with Rag1-, IL-4-, IFN-γ-, STAT4-, and STAT6-deficient mice were used as a source of antigen-specific T cells (Murphy et al., 1990; Ouyang et al., 1998; Shoemaker et al., 2006) and were bred and maintained under SPF conditions at the NIMR, London, Home Office, UK, Animals (Scientific Procedures) Act 1986 or at the Washington University School of Medicine. Female mice were used at 8–12 weeks old, and animal protocols were approved according to the Animals (Scientific Procedures) Act 1986, Home Office, UK. Reagents, including antibodies for T cell and DC preparation, purification and culture, media, cytokines, and cytokine mAbs have been described (Hosken et al., 1995; Shoemaker et al., 2006; Veldhoen et al., 2009; Veldhoen et al., 2006). LPS (*S. minnesota*) was from Alexis, chicken ovalbumin protein (OVA protein) was from from Sigma-Aldrich, and ovalbumin peptide_323-339_ (OVA) (endotoxin-free) was from Biosynthesis. U0126 was from BioMol International. PD184352 (MEK inhibitors), SB203580 (p38 inhibitor), and CT99021 (GSK3β inhibitor) were kind gifts from P. Cohen and N. Shpiro, University of Dundee, UK.

### Isolation of CD4^+^ T Cells and of Splenic DC and Cell Culture for T Cell Phenotype Differentiation

T cells were sorted for CD4^+^CD62L^hi^, CD4^+^CD62L^hi^CD25^−^, or CD4^+^CD44^lo^CD25^−^ to >98% on a Moflo cytometer (Cytomation) as before (Shoemaker et al., 2006; Veldhoen et al., 2009). In most cases, experiments were reproduced with each type of purified CD4^+^ T cell population with similar results obtained. Splenic DCs were prepared as described (Hosken et al., 1995), and sort purified CD11c^+^ cells were added to the T cell culture. Purified DO11.10 CD4^+^ T cells (1 × 10^5^ cells/ml) were cultured as before (Hosken et al., 1995), in a total volume of 1 ml cRPMI medium in a 48-well plate, with splenic DCs (2 × 10^4^ cells/ml), and varying amounts of OVA and of IL-12. APC-independent differentiation of naive CD4^+^ T cells into Th1 and Th2 cells used stimulation with anti-CD3 and anti-CD28 and appropriate cytokine conditions, and control Th1 and Th2 cells were cultured as described before (Hosken et al., 1995; Shoemaker et al., 2006). Culture conditions for Th17 cells were as described before (Veldhoen et al., 2006). Importantly, Th1 and Th2 cells could be differentiated in cRPMI or IMDM (Hosken et al., 1995; Shoemaker et al., 2006; Veldhoen et al., 2006), but Th17 cells were only differentiated optimally in IMDM (Veldhoen et al., 2009). When indicated, U0126 or PD184352 (MEK inhibitors), SB203580 (p38 inhibitor), CT99021 (GSK3β inhibitor), or a similar amount of DMSO were present in the culture. More details of specific culture conditions are provided in Figures S6 and S7.

### Cytokine Detection by ICS and ELISA

At day 5 or 7, cells were restimulated with immobilized anti-CD3 (2 μg/ml) and anti-CD28 (2 μg/ml) (4 hr with BrefeldinA [10 μg/ml] in the last 2 hr) or with PdBU and Ionomycin (5 hr with BrefeldinA [1 μg/ml]). After ICS FACS, data were collected on a FACSCalibur (Becton Dickinson) and analyzed with FlowJo (Tree Star). For ELISA, cells were similarly restimulated for 48 hr and supernatant was collected and analyzed for IL-4, IL-10, and IFN-γ as described before (Shoemaker et al., 2006).

### In Vivo Studies

BALB/c mice were injected intravenously (i.v.) with a red blood cell-depleted single-cell spleen suspension (2.5 × 10^7^ cells) (Castro et al., 2000) from DO11.10 WT or STAT-deficient mice. After 48 hr, they were injected subcutaneously with PBS or with OVA protein (5 mg) plus LPS (5 μg). The inguinal lymph nodes were removed 48 hr later. A single-cell suspension (1 × 10^6^ cells) was restimulated for 24 or 48 hr with 1 μM or 3 μM of OVA and with BrefeldinA for the last 6 hr. Half of the supernatant was removed before the addition of BrefeldinA for use in an ELISA assay. The cells were fixed and stained as before. Those positive for KJ1-26-Bio and for CD4-PerCP were gated, and IFN-γ and IL-10 staining was examined for this population and analyzed as before.

### Real-Time Quantitative RT-PCR

Cells were harvested and restimulated in the presence of immobilized anti-CD3 (2 μg/ml) plus anti-CD28 (2 μg/ml) for 3 hr or immediately lysed. RNA was extracted and reverse-transcribed and cDNA was analyzed for the expression of cytokines and transcription factors by real-time PCR assay as before (Shoemaker et al., 2006). Target gene mRNA expression was quantified either with SYBR Green (Applied Biosystems) or with Master Mix (Applied Biosystems) and normalized to *ubiquitin* or *HPRT* mRNA levels, respectively.

### Immunoblotting

Differentiated CD4^+^ T cells were rested for 5 hr in 1% FCS-containing medium and restimulated as described for specific experiments. Cell lysates were prepared, equal amounts of protein were separated by SDS-PAGE, and phosphorylated or total ERK and actin were detected as described before (Beinke et al., 2004).

## Supplemental Data

Document S1. One Table, Five Figures, and Two Experimental Flowcharts
