# Supplementary material for: Interleukin-10 Production by Th1 Cells Requires Interleukin-12-Induced STAT4 Transcription Factor and ERK MAP Kinase Activation by High Antigen Dose
Source: Immunity. 2009 Aug 21;31(2-3):209–19. doi: 10.1016/j.immuni.2009.05.012 (PMC2791889; doi:10.1016/j.immuni.2009.05.012)
Supplement: Document S1. One Table, Five Figures, and Two Experimental Flowcharts [file mmc1.pdf]

Immunity, Volume *31*

Supplemental Data

Interleukin-10 Production by Th1 Cells Requires Interleukin-12-Induced STAT4 Transcription Factor and ERK MAP Kinase Activation by High Antigen Dose

Margarida Saraiva, Jillian R. Christensen, Marc Veldhoen, Theresa L. Murphy, Kenneth M. Murphy, and Anne O'Garra

**Table S1. Cellular fold recovery after 7 days culture**			
**0.7 µM**	**0.7 µM + IL-12**	**0.01/0.05 µM**	**0.01/0.05 µM + IL-12**
35	20	6.7	2.3
35.2	22	9.2	6
74.6	30.9	14.1	4.6
46	28	5.5	2.6

Table S1. Cellular fold recovery after 7 days culture.

$CD4^+$ T cells from DO11.10 TCR transgenic animals were isolated and differentiated for 7 days as indicated and the fold recovery determined for various experiments performed. Mean±SD is indicated from results of 3 separate experiments.

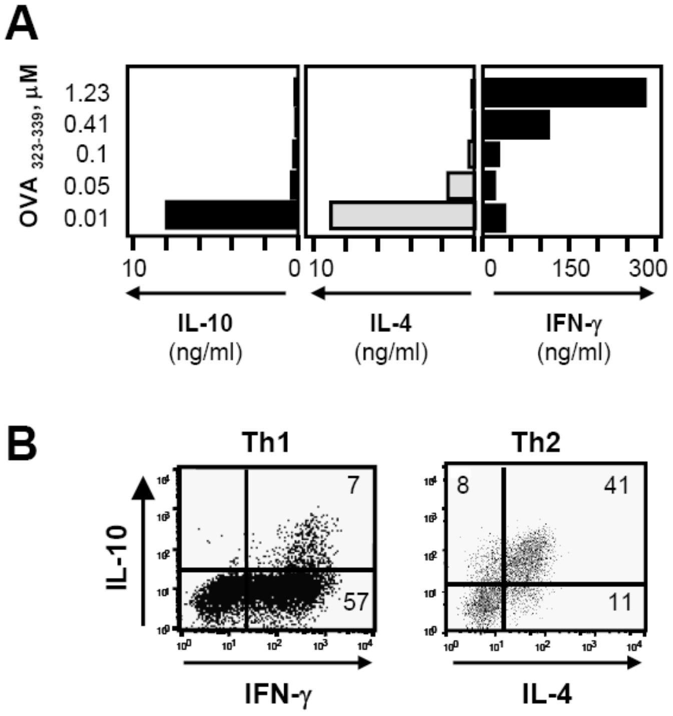

Figure S1. High antigen dose-induced Th1 cells do not express IL-10, but IL-12-induced Th1 cells in an APC-free system do.

(A) $CD4^+$ T cells from DO11.10 TCR transgenic animals were isolated and differentiated for 7 days with splenic DC, in medium containing increasing doses of OVA as indicated. On day 7, cells were harvested and restimulated for 48h in the presence of plate bound anti-CD3 and soluble anti-CD28. The culture supernatants were analyzed by ELISA for the production of IL-10, IFN-γ and IL-4. (B) $CD4^+$ T cells were cultured for 1 week in the presence of plate bound anti-CD3 soluble anti-CD28 with IL-12 and anti-IL-4 antibody (Th1) or with IL-4 (Th2). On day 7, cells were restimulated for 4h in the presence of BrefeldinA and stained at the single cell level for IL-10, IFN-γ and IL-4. Data is representative of 10 experiments performed.

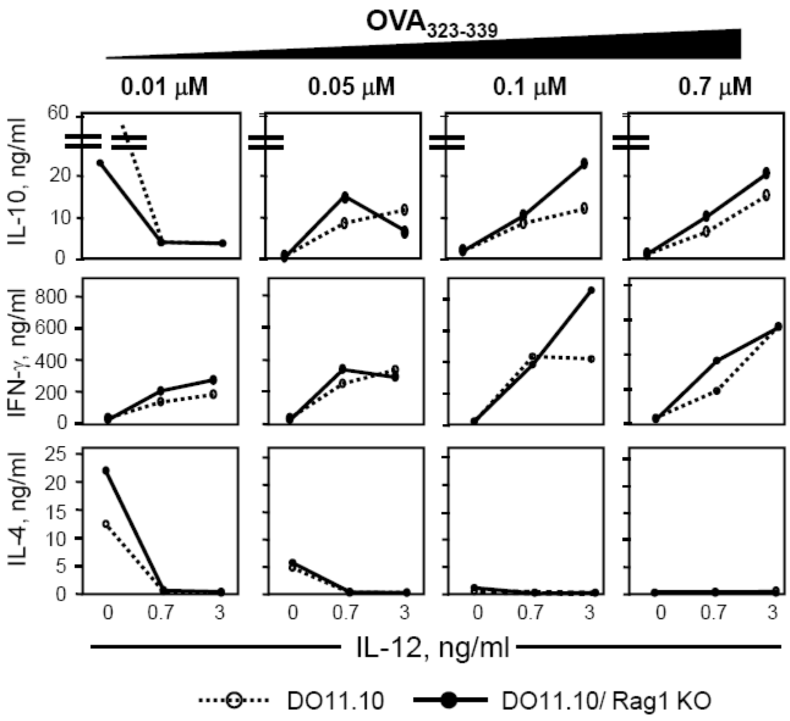

Figure S2. Induction of IL-10 in Th1 cells by IL-12 at high antigen dose is not dependent on effector cells.

$CD4^+$ T cells from DO11.10 TCR transgenic or DO11.10/Rag1 deficient mice were isolated, differentiated for 7 days and restimulated as in Figure 1. Cytokine production was detected by ELISA and representative results are shown from two different experiments.

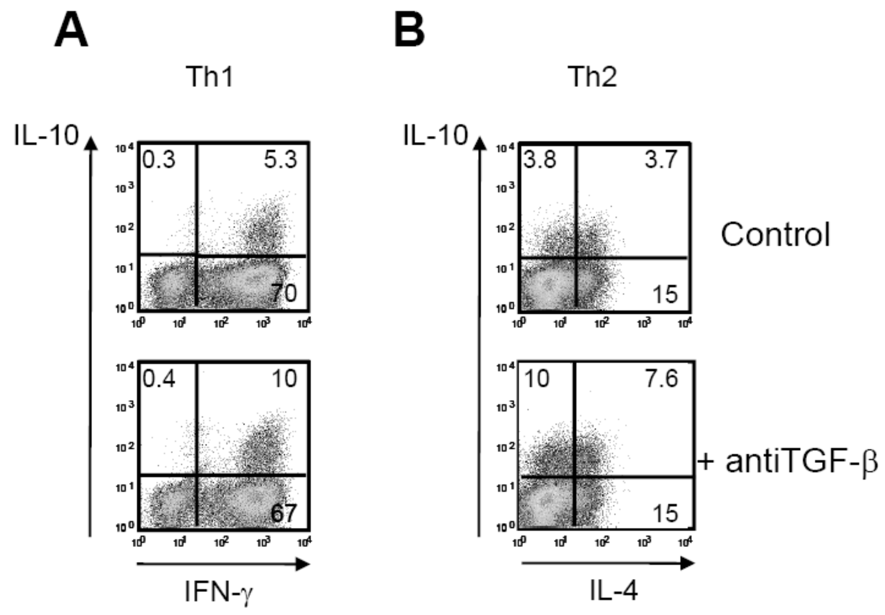

Figure S3. Neutralization of TGF-β enhances IL-10 production by both Th1 and Th2 cells.

$CD4^+$ T cells were isolated and cultured for 5 days in the presence of plate bound anti-CD3, soluble anti-CD28 and IL-12 (3ng/ml) plus anti-IL-4 (20μg/ml), Th1; or IL-4 (10ng/ml) plus anti-IL-12 (10μg/ml), Th2; in the absence (Control) or presence of a TGF-β neutralizing antibody (10μg/ml; + anti-TGF-β). On day 5, cells were restimulated with PdBU and Ionomycin and the expression of IL-10, IFN-γ and IL-4 was detected by ICS. Representative results are shown from 4 experiments.

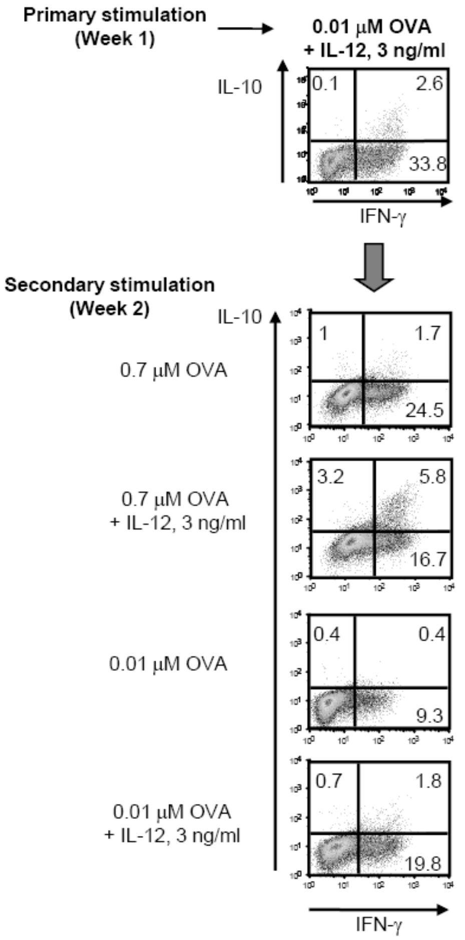

Figure S4. Repeated low antigen dose stimulation does not give rise to IL-10 expression by Th1 cells, but can be rescued by high antigen dose and IL-12.

$CD4^+$ T cells from DO11.10 TCR transgenic animals were cultured for 1 week in the presence of low (0.01μM) dose of OVA and DC plus IL-12, and then harvested, counted and re-cultured for a second week as indicated. At the end of the first and the second weeks, cells were restimulated with anti-CD3 and anti-CD28 and IFN-γ and IL-10 cytokine expression detected by ICS, as described before. Representative results are shown from 2 different experiments.

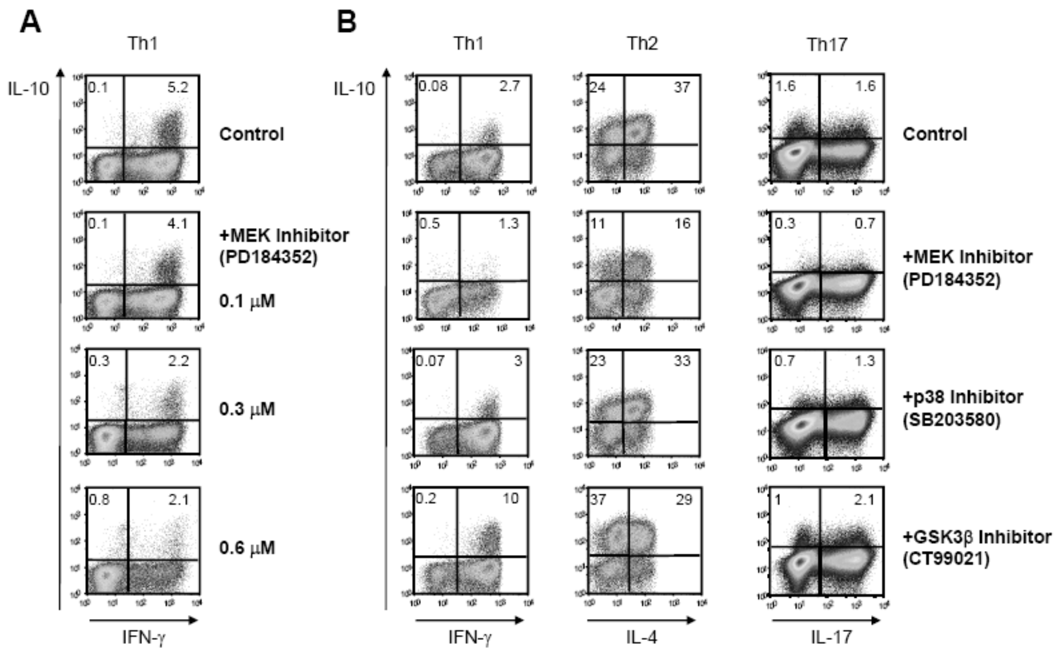

Figure S5. IL-10 production by Th1, Th2 and Th17 cells is inhibited by the MEK inhibitor, but not by a p38 inhibitor, nor a GSK3β inhibitor.

$CD4^+$ T cells were isolated and cultured for 7 days in the presence of plate bound anti-CD3, anti-CD28 and IL-12 (3ng/ml), Th1; IL-4 (10ng/ml) plus anti-IFN-γ (10μg/ml), Th2; or IL-6 (50ng/ml), TGF-β (1ng/ml) and IL-1 (10ng/ml), Th17; all in the absence (Control) or presence of PD184352 MEK Inhibitor (0.1, 0.3, 0.6μM in A and 1μM in B), SB203580 p38 Inhibitor (1μM) or CT99021 GSK3β Inhibitor (1μM). On day 5 or 7, cells were restimulated and the expression of IL-10, IFN-γ, IL-4 and IL-17 was detected by ICS.

Experimental flowchart used for Figures 1, 2 and 6A and 6B and Figures S1 and S2

Balb/c
CD11c^{bright} Splenic (sp) DC
MACS enriched
Sort purified

Balb/c
CD11c^{bright} Splenic (sp) DC
MACS enriched
Sort purified

↓

1x10^5 T cells; 2x10^4 spDC
Co-cultured in 48 well-plates, in complete RPMI
Increasing doses of Ovalbumin peptide 323-339 (OVA)
Medium or 3 ng/ml of IL-12

↓

Day 4: split cells

↓

Day 7:
-harvest cells

- restimulate with 2 µg/ml of plate-bound αCD3 and 2 µg/ml of soluble αCD28 for 4h, the last 2h in the presence of brefeldin A; cytokine production analysed by ICS
- restimulate with 2 µg/ml of plate-bound αCD3 and 2 µg/ml of soluble αCD28 for 3h (RNA) or 24h (ELISA)

Experimental flowchart used for Figure 4 and Figure S4

Balb/c WT or Il12b$^{-/-}$
CD11c^{bright} sp DC
MACS enriched
Sort purified

Balb/c-DO11.10 WT
CD4$^+$T cells
Sort purified

↓

1x10^{5} T cells; 2x10^4 spDC
Co-cultured in 48 well-plates, in complete RPMI
Increasing doses of OVA
Medium or 3 ng/ml of IL-12

↓

Day 4: split cells

↓

Day 7:
-harvest cells

- restimulate with 2 µg/ml of plate-bound αCD3 and 2 µg/ml of soluble αCD28 for 4h, the last 2h in the presence of brefeldin A; cytokine production analysed by ICS

RECULTURE WITH SORT PURIFIED sp DC FOR A SECOND WEEK as above but also with IL-2

Low antigen dose-driven Th2: 0.05 µM OVA; no IL-4 * IL-4-driven Th2: IL-4 at 10 ng/ml; αIL-12 at 10 µg/ml; 1 µM OVA

Experimental flowchart used for Figures 5C and 5D, 6C and 6D and Figures S3 and S5

T cells
CD4$^+$
Sort purified

↓

0.5x10^6 T cells cultured in 48 well-plates, in complete RPMI or IMDM
Plate bound αCD3 and αCD28
DMSO or Inhibitors (at 0.5 or 1 μM) or αTGF-β (at 10 μg/ml)
Th1; Th2 or Th17 differentiation conditions*

Th1 and Th2

Day 3: split cells

Day 7:

Th17

Day 5:

-harvest cells
- restimulate with PdBU and Ionomycin for 5h, the last 2h in the presence of brefeldin A; cytokine production analysed by ICS

* Th1: IL-12 at 3 ng/ml;
* Th2: IL-4 at 10 ng/ml;
* Th17: IL-6 at 50 ng/ml; TGF-β at 1 ng/ml; IL-1 at 10 ng/ml

Supplementary Figures 6 and 7: Schematic representation of the experimental design used in this work.
